# Crepidatumines C and D, Two New Indolizidine Alkaloids from *Dendrobium crepidatum* Lindl. ex Paxt.

**DOI:** 10.3390/molecules24173071

**Published:** 2019-08-23

**Authors:** Xiaolin Xu, Zesheng Li, Runmei Yang, Houguang Zhou, Yanbin Bai, Meng Yu, Gang Ding, Biao Li

**Affiliations:** 1 Key Laboratory of Bioactive Substances and Resources Utilization of Chinese Herbal Medicine, Ministry of Education, Institute of Medicinal Plant Development, Chinese Academy of Medical Sciences and Peking Union Medical College, Beijing 100193, China; 2 Yunnan Dehong Institute of Tropical Agricultural Science, Dehong 678600, China

**Keywords:** *Dendrobium crepidatum* Lindl. ex Paxt., indolizidine alkaloids, crepidatumines, structure elucidation, hypoglycemic effect

## Abstract

Two new indolizidine alkaloids, crepidatumines C (**1**) and D (**2**), together with crepidine (**3**), isocrepidamine (**4**), and crepidamine (**5**) were isolated from the *Dendrobium crepidatum* Lindl. ex Paxt. X-ray diffraction experiments established the absolute configurations of known compounds **3** and **4**. The planar structures and relative configurations of new compounds **1** and **2** were elucidated by extensive spectra analysis including HR-ESI-MS, NMR (^1^H, ^13^C, ^1^H-^1^H COSY, HSQC, HMBC, and NOESY spectra), and the absolute configurations of **1** and **2** were suggested on the basis of possible biosynthetic pathways. The biological results confirmed that isocrepidamine (**4**) displayed a potent hypoglycemic effect in vitro without cytotoxicity.

## 1. Introduction

**Figure 1:**
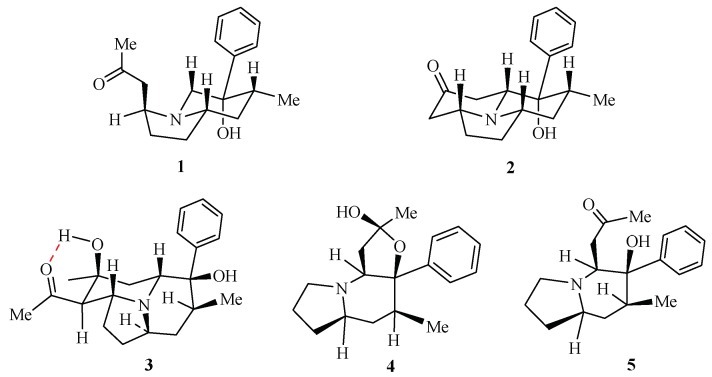
Structures of **1**–**5**.

*Dendrobium*, a genus of Orchidaceae, is distributed in south of China [1,2]. The stems of several *Dendrobium* species are used as precious traditional Chinese medicines with the effect of maintaining gastric tonicity, enhancing the production of body fluids, and relieving and curing symptoms of dryness and body heat [3]. *Dendrobium crepidatum* Lindl. ex Paxt. Is considered as one of the sources of “Shi-Hu”. It produces indolizine-type alkaloids, and so far, only several indolizine-type alkaloids and two stilbene derivatives have been isolated from this medicinal plant [4,5,6,7,8,9]. Two indolizidine alkaloids, crepidatumines A and B, with novel skeletons, together with the stereoisomer of dendrocrepidine B and dendrocrepine, were isolated during our previous chemical investigation of this medicinal plant [10]. Actually, the biosynthesis of indolizidine alkaloids from the *Dendrobium crepidatum* Lindl. ex Paxt. is still not clear. In order to obtain the potential intermediates or shunt products of the biosynthesis, five indolizidine alkaloids including the two new analogs crepidatumines C (**1**) and D (**2**) together with crepidine (**3**), isocrepidamine (**4**), and crepidamine (**5**) were isolated from the total alkaloid extract [5]. (Figure 1) In this paper, the structure elucidation, biological evaluation, and possible biogenetic origin of compounds **1**–**5** arereported.

## 2. Results and Discussion

**Figure 2:**
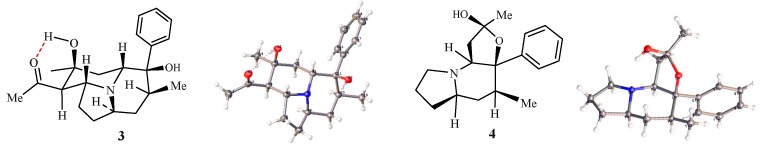
X-ray crystal structure of **3** and **4**.

The structures of compounds **3** and **4** were determined to be crepidine and isocrepidamine, which were supported by X-ray diffraction experiments (Figure 2), and compound **5** was characterized to be crepidamine based on the NMR data [5].

**Figure 3:**
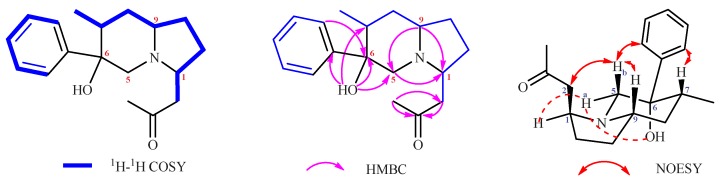
^1^H-^1^H COSY, HMBC, and NOESY correlations of **1**.

The molecular formula of **1** was determined to be C_18_H_25_NO_2_ based on the HRESIMS (*m/z* 288.1968 [M + H]^+^, calcd 288.1964). The ^1^H, ^13^C and HMQC spectra of **1** (Table 1) revealed the presence of two methyls, five methylenes, three methines, an oxygenated carbon with chemical shift value δ_C_ = 76.5, a keto group with chemical shift value δ_C_ = 206.8, and a mono-substituted phenyl ring. In addition, a singlet proton formed as a free hydroxyl or amino group.The ^1^H-^1^H COSY correlations established three isolated spin-systems including a mono-substituted phenyl unit, and a fragment: –C-12–C-7–C-8–C-9–C-10–C-11–C-1–C-2–. Analysis of HMBC correlations elucidated the structure of **1** (Figure 3). The HMBC cross peaks from 6-OH to C-5, C-6, C-7, and C-1′ determined the direct connectivities of C-6 with C-5, C-7, and C-1′ with a hydroxyl group anchored at C-6; the correlations of CH_3_-4 with C-2 and C-3 established the linkage of CH_3_-4 with C-2 and C-3; and correlations of CH_2_-5 with C-1 and C-9, and H-9 with C-1, established an indolizidine ring system. Thus, the planar structure of **1** was characterized. The relative configuration of **1** was determined on the basis of analysis of NOESY correlations. The NOESY correlations from H-5b, H-7 to H-2′ (H-6′), and from H-5b to H-9 confirmed that these protons or groups were on the same side of the corresponding piperidine ring; the correlations of –CH_2_-2 with H-5b, and of H-1 and 6-OH with H-5a demonstrated that these protons were close to each other in space. Thus, the relative configuration of **1** was determined (Figure 3).

**Figure 4:**
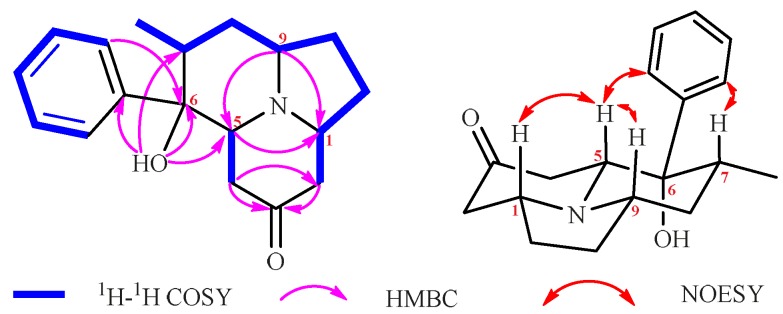
^1^H-^1^H COSY, HMBC, and NOESY correlations of **2**.

The HRESIMS (*m/z* 286.1809 [M + H]^+^, calcd 286.1807) assigned the molecular formula of **2** as C_18_H_23_NO_2_. The ^1^H, ^13^C and HMQC spectra of **2** (Table 1) revealed the presence of one methyl, five methylenes, four methines, an oxygenated carbon with chemical shift value δ_C_ = 76.5, a keto group with chemical shift value δ_C_ = 208.5, and a mono-substituted phenyl ring. In addition, there are two singlet protons as free hydroxyl or amino groups. These data accounted for all the ^1^H and ^13^C-NMR resonances together considering the degrees of unsaturation, implying that **2** possessed a tricyclic system. The ^1^H-^1^H COSY correlations established three isolated proton spin-systems including a mono-substituted phenyl unit, and two fragments: –C-12–C-7–C-8–C-9–C-10–C-11–C-1–C-2– and –C-4–C-5–. Analysis of HMBC correlations elucidated the structure of **2** (Figure 4). Correlations of H-5 with C-1 and C-9, and H-9 with C-1 established an indolizidine ring system. The HMBC cross peaks from 6-OH to C-5, C-6, C-7, and C-1′ determined the direct connectivities of C-6 with C-5, C-7, and C-1′ with a hydroxyl group anchored at C-6.The correlations of H-4 with C-2 and C-3 established the linkage of C-4 with C-2 and C-3. Thus, the planar structure of **2** was characterized. The relative configuration of **2** was determined on the basis of analysis of NOESY correlations. The correlations from H-5 with H-1, H-7, H-9 and 7-OH implied that these groups possessed β-configurations. The NOESY correlations from 12-Me to H-2′ (H-6′) confirmed that 12-Me and the mono-substituted phenyl group were on the same side of the corresponding furan ring. Thus, the relative configuration of **2** was determined (Figure 4).

**Figure 5:**
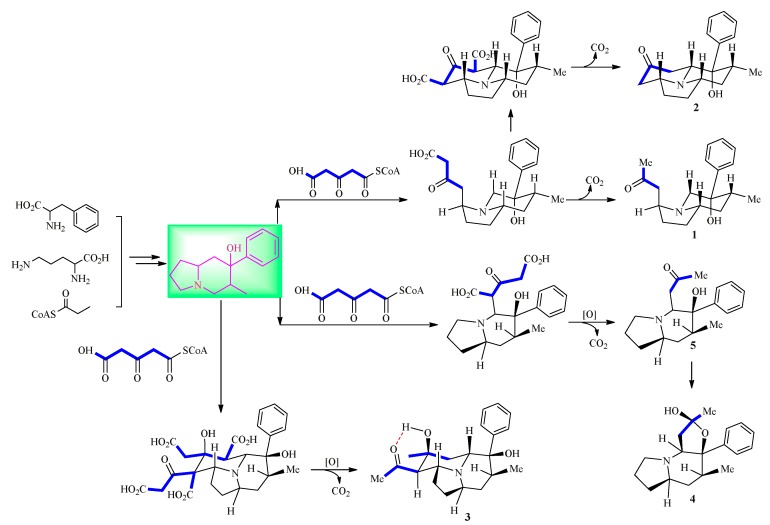
The possible biosynthetic pathway of compounds **1**–**5**.

The absolute configurations of **1** and **2** were suggested to be same as those of **3**–**5** on the basis of similar biosynthesis pathway (Figure 5, Supplementary Materials). Structures of **3** and **4** were determined by the X-ray diffraction experiments, and showed that **4** is a racemate.

**Figure 6:**
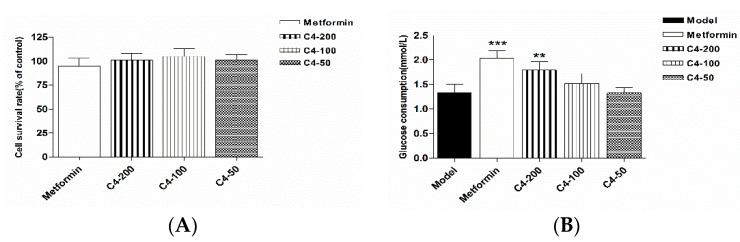
Effect of compound 4 (C4) on cell viability (**A**) and glucose consumption (**B**) in the HepG2 cell. Data are shown as the mean ± SD (*n* = 6). ** *p* < 0.01, *** *p* < 0.001 versus model.

The hypoglycemic effect of compound **4** (isocrepidamine) was evaluated using the high glucose model of HepG2 cells. As a result, at the concentrations of 200 μmol/L, this compound significantly increased the glucose consumption by 34% compared with the model group, which hadnon-cytotoxicity as per the cell counting kit-8 (CCK-8) assay (Figure 6).

## 3. Experimental Section

### 3.1. General Experimental Procedures

Optical rotations were measured on a PerkinElmer 241 polarimeter (Perkin Elmer, Inc., Waltham, MA, USA), and UV data were determined on a ThermoGenesys-10S UV-vis spectrometer (Fisher Scientific, Illkirch, France). IR data were recorded using a Nicolet IS5FT-IR spectrophotometer (Shimadzu, Kyoto, Japan). CD spectra were obtained on a JASCO J-810 spectrometer (JASCO, Tokyo, Japan). ^1^H and ^13^C-NMR data were acquired with a Bruker 600 spectrometer (Bruker, Rheinstetten, Germany) using solvent signals (DMSO-*d*_6_; *δ*_H_ 2.50/*δ*_C_ 39.5) as references. The HMQC and HMBC experiments were optimized for 145.0 and 8.0 Hz, respectively. HRESIMS were obtained using a TOF-ESI-MS (Waters Synapt G2, Milford, MA, USA). Semipreparative HPLC separation was carried out using a Lumtech instrument packed with a YMC-Pack ODS-A column (YMC Co., Ltd., Kyoto, Japan, 5 μm, 250 × 10 mm). Sephadex LH-20(Pharmacia Biotech AB, Uppsala, Sweden) and silica gel (200–300 mesh) (Qingdao Marine Chemical Plant, Qingdao, China) were used.

### 3.2. Plant Materials

The stems of *Dendrobium crepidatum* Lindl. ex Paxt. were collected from Ruili Resource Nursery of Dendrobium Germ Plasm and Resources, the Ministry of Agriculture and Rural Affairs of the People’s Republic of China (Yunnan, China) in August 2017. The sample was identified by one of the co-authors Ze-Sheng Li from Yunnan Dehong Institute of Tropical Agricultural Science (Yunnan, China). A voucher specimen was deposited in the herbarium of the Institute of Medicinal Plant Development, Chinese Academy of Medical Sciences (Beijing, China).

### 3.3. Extraction and Isolation

The dried stems of *Dendrobium crepidatum* Lindl. ex Paxt. (9.0 kg) were extracted under reflux with 95% ethanol (50 L × 3 h, three times). The combined extract was suspended with water, and extracted with petroleum ether and CH_2_Cl_2_ three times separately. The fraction of CH_2_Cl_2_ was concentrated into extracts, and dissolved in 5% hydrochloric acid filtered, then adjusted to pH 10 with ammonia water. Finally, it was extracted by CH_2_Cl_2_ three times at room temperature. The CH_2_Cl_2_ extract was obtained the total alkaloids 90 g of crude extract. The original extract was fractionated on a silica gel CC eluted with petroleum ether- acetone (50:1, 40:1, 30:1, 20:1, 15:1, 10:1, 5:1, 2:1 and 0:1, *v*/*v*, each 6.6 L) to give five fractions (Fr.1 to Fr.5). Fr.2 (10 g) was fractionated on a silica gel column chromatography (CC) using petroleum ether-acetone isocratic elution (30:1) to afford six fractions (Fr.2.1–Fr.2.6). Fr.2.1 (10.0 g) was purified by semi-preparative HPLC (60–100% MeOH-H_2_O for 30.0 min, *v*/*v*, 2 mL/min) to obtain crepidine (**3**; 105 mg, *t*_R_ 29.0 min), and isocrepidamine (**4**; 2.0 g, *t*_R_ 32.7 min). Separation of Fr.2.4 (2.0 g) was performed over Sephadex LH-20 (CH_2_Cl_2_: MeOH/1:1) to give four fractions (Fr.2.4.1–Fr.2.4.4). Fr.2.4.2 (500 mg) was further purified by semi-preparative HPLC (60–100% MeOH-H_2_O for 30 min, *v*/*v*, 2 mL/min) to obtain crepidamine (**5**; 30.0 mg, *t*_R_ 21.0 min). Fr.2.4.3 (1.0 g) was purified by semi-preparative HPLC (60–100% MeOH-H_2_O for 30.0 min, *v*/*v*, 2 mL/min) to obtain crepidatumine C (**1**; 4.0 mg, *t*_R_ 32.8 min), and crepidatumine D (**2**; 25.0 mg, *t*_R_ 24.7 min).

Compound **1**: white powder; [α]_D_^25^−3.00 (c 0.1, MeOH); UV (MeOH) λ_max_ (log ε) 206 (3.68); IR (neat) υ_max_ 2930, 1714, 1036, 766, 704 cm^−1^; for ^1^H-NMR and ^13^C-NMR data see Table 1; Positive HR-ESI-MS: *m*/*z* 288.1964 (calcd. for C_18_H_26_NO_2_ [M + H]^+^, 288.1968).

Compound **2**: white powder; [α]_D_^25^−4.00 (c 0.1, MeOH); UV (MeOH) λ_max_ (log ε) 209 (3.81); IR (neat) υ_max_ 3482, 2964, 1708, 999, 776, 709 cm^−1^; for ^1^H-NMR and ^13^C-NMR data see Table 1; Positive HR-ESI-MS: *m*/*z* 286.1807 (calcd. for C_18_H_24_NO_2_ [M + H]^+^, 286.1809).

### 3.4. X-Ray Crystallographic Analysis of **3** and **4**.

Upon crystallization from *n*-Hexane–CH_2_Cl_2_ (10:1) using the vapor diffusion method, colorless crystals were obtained for **3**. C_21_H_29_NO_3_, M = 343.45, orthorhombic, a = 5.7476(3) Å, b = 17.5786(5) Å, c = 17.7942(6) Å, U = 1797.84(11) Å3, T = 109.1(3), space group P212121 (No. 19), Z = 4, μ(Cu Kα) = 0.666, 9652 reflections measured, 3383 unique (Rint = 0.0609), which were used in all calculations. The final wR (F2) was 0.1367 (all data).

Crystallographic data for the structure of **3** has been deposited in the Cambridge Crystallographic Data Centre (deposition number: CCDC 1936544) (Table 2).

Upon crystallization from *n*-Hexane–CH_2_Cl_2_ (10:1) using the vapor diffusion method, colorless crystals were obtained for **4**. C_20_H_27_NO_4_, M = 345.42, orthorhombic, a = 6.6679(4) Å, b = 10.7681(4) Å, c = 24.2111(9) Å, U = 1738.38(13) Å3, *T* = 107.75(10), space group P2_1_2_1_2_1_ (no. 19), Z = 4, *μ* (Cu Kα) = 0.737, 5698 reflections measured, 3256 unique (Rint = 0.0271), which were used in all calculations. The final wR (F2) was 0.1106 (all data).Crystallographic data for the structure of **4** has been deposited in the Cambridge Crystallographic Data Centre (deposition number: CCDC 1908235) (Table 3).

### 3.5. In Vitro Evaluation of Compound **4**

Cell culture: Human hepatoma cells HepG2 were cultured in Dulbecco’s modified Eagle’s medium (DMEM, HyClone). The medium was supplemented with 10% fetal bovine serum (Gibco) and 1% penicillin/streptomycin (HyClone) in a humidified atmosphere of 5% CO_2_ and 37 °C.

Assay for cell viability: The assay for cell viability was determined with the cell counting kit-8 (CCK-8). HepG2 cells were seeded in 96-well plates as 2.5 × 10^3^ cells each well. After culturing for 24 h, the control group was added with serum-free medium, while the experimental groups were with the medium containing different concentrations (50, 100, and 200 μmol/L) of compound **4** or 200 μmol/L of metformin for another 24 h. Then the cells were treated with CCK-8 for 4 h. Finally, the absorbance was measured at 450 nm. The cell survival rate was calculated as the absorbance of each treated well divided by the control.

Assay for hypoglycemic activity: For the experiment, the cells were seeded in 96-well plates as 1 × 10^4^ cells each well. After culturing for 24 h, the medium containing different concentrations (50, 100 and 200 μmol/L) of compound **4** was added for 24 h. The cells with 200 μmol/L metformin treatment were taken as positive control and the cells with phenol red-free DMEM as control. After the drug treatment, the glucose concentrations of the medium were determined with the glucose oxidase method. The glucose consumption of each well was obtained by subtracting the glucose concentrations of the experimental medium from the control group.

## 4. Conclusions

Two new indolizidine alkaloids crepidatumines C (**1**) and D (**2**) together with crepidine (**3**), isocrepidamine (**4**), and crepidamine (**5**) were isolated from the *Dendrobium crepidatum* Lindl. ex Paxt., and their structures were determined by HR-ESI-MS, NMR (^1^H, ^13^C, ^1^H-^1^H COSY, HSQC, HMBC, and NOESY spectra), and X-ray diffraction experiments. The results enrich the chemical diversity and further provide the key intermediates in the biosynthetic pathway of indolizidine alkaloids from *Dendrobium crepidatum* Lindl. ex Paxt., implying that more minor intermediates or shunt products might exist in the medicinal plants. In addition, the biological study showed a potent hypoglycemic effect of isocrepidamine (**4**) in vitro without cytotoxicity.

## Figures and Tables

**Table 1:** NMR spectroscopic data of **1** and **2** in (DMSO-*d*_6_) (*δ* in ppm and *J* in Hz) ^a,b^.

Pos	1		2	
	δ_C_ *^b^*, Type	δ_H_ *^a^*, mult. (*J* in Hz)	δ_C_ *^b^*, Type	δ_H_ *^a^*, mult. (*J* in Hz)
1	74.8, CH	4.10, m	59.9, CH	3.37, m
2	48.6, CH_2_	2.46, m	44.1, CH_2_	2.33, dd (10.8, 13.8) 1.92, ddd (1.8, 4.2, 13.8)
3	206.8, qC		209.6, qC	
4	30.9, CH_3_	2.07, s	38.6, CH_2_	1.13, ddd (1.8, 3.0, 13.2) 2.63, t (13.2)
5a 5b	67.1, CH_2_	2.95, d (10.8) 2.69, d (10.8)	66.7, CH	3.00, dd (3.0, 13.2)
6	76.5, qC		76.2, qC	
7	38.4, CH	1.96, m	31.7, CH	2.46, m
8	36.3, CH_2_	1.49, m	35.5, CH_2_	1.46, m 1.74, dt (3.0, 11.4)
9	63.9, CH	2.43, m	51.4, CH	3.15, m
10	30.2, CH_2_	1.65, m 1.46, m	29.7, CH_2_	1.40, m 1.97, m
11	30.8, CH_2_	1.67, m 1.25, m	29.1, CH_2_	1.39, m 2.04, m
12	14.4, CH_3_	0.48, d (6.6)	16.1, CH_3_	0.71, d (6.6)
1′	146.1, qC		143.9, qC	
2′/6′	128.2, CH	7.46, dd (1.2, 8.4)	126.9, CH	7.41, br d (7.2)
3′/5′	125.6, CH	7.31, br d (8.4)	128.2, CH	7.34, dd (7.2)
4′	126.8, CH	7.21, br t (8.4)	127.0, CH	7.24, dd (7.2)
6-OH		4.76, s		4.53, s

^a^ Assignments were based on HSQC, HMBC, and ^1^H-^1^H COSY experiments. ^b^ NMR spectroscopic data were recorded at 600 MHz (^1^H NMR), 150 MHz (^13^C NMR).

**Table 2:** Crystal data and structure refinement for **3.**

Identification Code	3
Empirical formula	C_21_H_29_NO_3_
Formula weight	343.45
Temperature/K	109.1(3)
Crystal system	orthorhombic
Space group	P2_1_2_1_2_1_
a/Å, b/Å, c/Å	5.7476(3), 17.5786(5), 17.7942(6)
α/°, β/°, γ/°	90, 90, 90
Volume/Å^3^	1797.84(11)
Z	4
ρ_calc_/mg mm^−3^	1.269
μ/mm^−1^	0.666
F (000)	744
Crystal size/mm^3^	0.35 × 0.12 × 0.02
2θ range for data collection	7.06 to 142.74°
Index ranges	−6 ≤ h ≤ 7, −18 ≤ k ≤ 21, −21 ≤ l ≤ 19
Reflections collected	9652
Independent reflections	3383[R(int) = 0.0609 (inf-0.9Å)]
Data/restraints/parameters	3383/0/231
Goodness-of-fit on F^2^	1.030
Final R indexes [I > 2σ (I) i.e., F_o_ > 4σ (F_o_)]	R_1_ = 0.0510, wR_2_ = 0.1302
Final R indexes [all data]	R_1_ = 0.0551, wR_2_ = 0.1367
Largest diff. peak/hole/e Å^−3^	0.275/−0.288
Flack Parameters	0.2(2)
Completeness	0.993

**Table 3:** Crystal data and structure refinement for compound **4**.

Identification Code	4
Empirical formula	C_20_H_27_NO_4_
Formula weight	345.42
Temperature/K	107.75(10)
Crystal system	orthorhombic
Space group	P2_1_2_1_2_1_
a/Å, b/Å, c/Å	6.6679(4), 10.7681(4), 24.2111(9)
α/°, β/°, γ/°	90, 90, 90
Volume/Å^3^	1738.38(13)
Z	4
ρ_calc_/mg mm^−3^	1.320
μ/mm^−1^	0.737
F (000)	744
Crystal size/mm^3^	0.350 × 0.340 × 0.100
2θ range for data collection	8.988 to 142.446°
Index ranges	−4 ≤ h ≤ 7, −11 ≤ k ≤ 13, −29 ≤ l ≤ 29
Reflections collected	5698
Independent reflections	3256[R(int) = 0.0271 (inf-0.9Å)]
Data/restraints/parameters	3256/0/230
Goodness-of-fit on F^2^	1.054
Final R indexes [I > 2σ (I) i.e., F_o_ > 4σ (F_o_)]	R_1_ = 0.0397, wR_2_ = 0.1072
Final R indexes [all data]	R_1_ = 0.0421, wR_2_ = 0.1106
Largest diff. peak/hole/e Å^−3^	0.312/−0.240
Flack Parameters	−0.13(14)
Completeness	0.9984

## Supplementary Materials

The following including NMR, IR, UV and HR-ESI-MS spectra of compounds **1** and **2** are available online.
