# Supplementary material for: Crepidatumines C and D, Two New Indolizidine Alkaloids from Dendrobium crepidatum Lindl. ex Paxt
Source: Molecules. 2019 Aug 23;24(17):3071. doi: 10.3390/molecules24173071 (PMC6749285; doi:10.3390/molecules24173071)
Supplement: Supplementary file 1 [file molecules-24-03071-s001.pdf]

molecules

Supplementary Material

Crepidatumines C and D, Two New Indolizidine Alkaloids from *Dendrobium crepidatum* Lindl. ex Paxt

Xiaolin Xu [1,†], Zesheng Li [2,†], Runmei Yang [1], Houguang Zhou [2], Yanbin Bai [2], Meng Yu [1], Gang Ding [1,*] and Biao Li [1,*]

[1] Key Laboratory of Bioactive Substances and Resources Utilization of Chinese Herbal Medicine, Ministry of Education, Institute of Medicinal Plant Development, Chinese Academy of Medical Sciences and Peking Union Medical College, Beijing 100193, China

[2] Yunnan Dehong Institute of Tropical Agricultural Science, Dehong 678600, China

* Correspondence: dgfyhchina@163.com or gding@implad.ac.cn (D.G.); bli@implad.ac.cn (B.L.); Tel.: +86-010-5783-3281 (D.G.)

† Theses authors contributed equally to this work.

Content

Experimental Section

Figure S1. ^{1}H NMR spectrum of compound **1**

Figure S2. ^{13}C NMR spectrum of compound **1**

Figure S3. HSQC spectrum of compound **1**

Figure S4. ^{1}H-^{1}H COSY spectrum of compound **1**

Figure S5. HMBC spectrum of compound **1**

Figure S6. NOESY spectrum of compound **1**

Figure S7. IR spectrum of compound **1**

Figure S8. UV spectrum of compound **1** in CH_3OH

Figure S9. HRESIMS spectrum of compound **1**

Figure S10. ^{1}HNMR spectrum of compound **2**

Figure S11. ^{13}C NMR spectrum of compound **2**

Figure S12. HSQC spectrum of compound **2**

Figure S13. ^{1}H-^{1}H COSY spectrum of compound **2**

Figure S14. HMBC spectrum of compound **2**

Figure S15. NOESY spectrum of compound **2**

Figure S16. IR spectrum of compound **2**

Figure S17. UV spectrum of compound **2** in CH3OH

Figure S18. HRESIMS spectrum of compound **2**

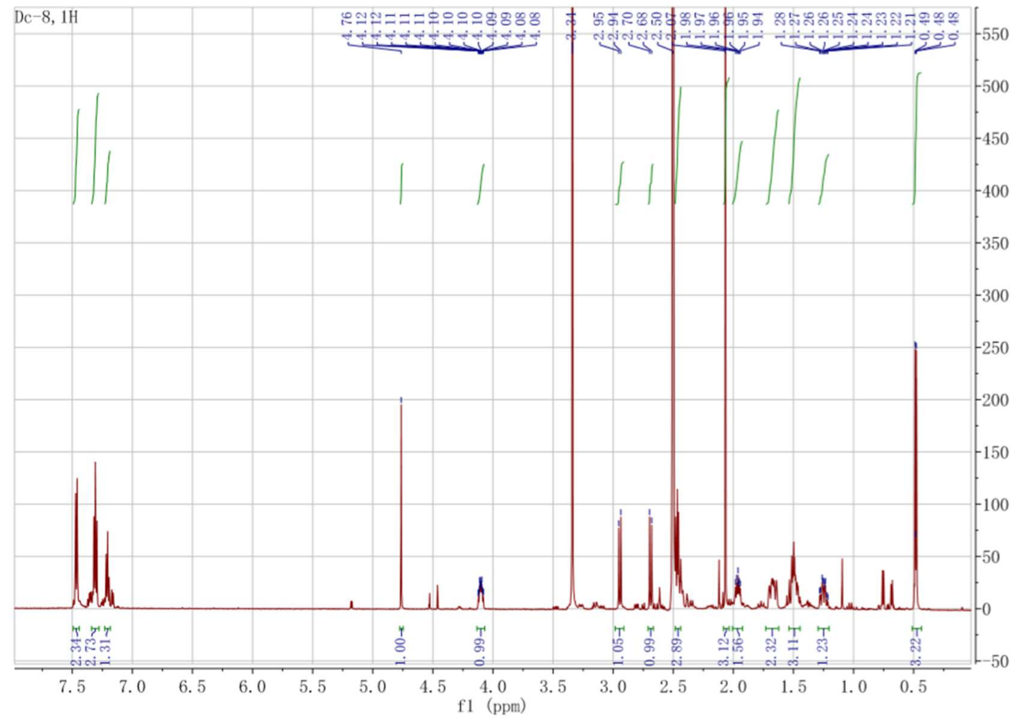

Figure 1. ^{1}H NMR spectrum of compound **1** (DMSO-d_6, 600MHz).

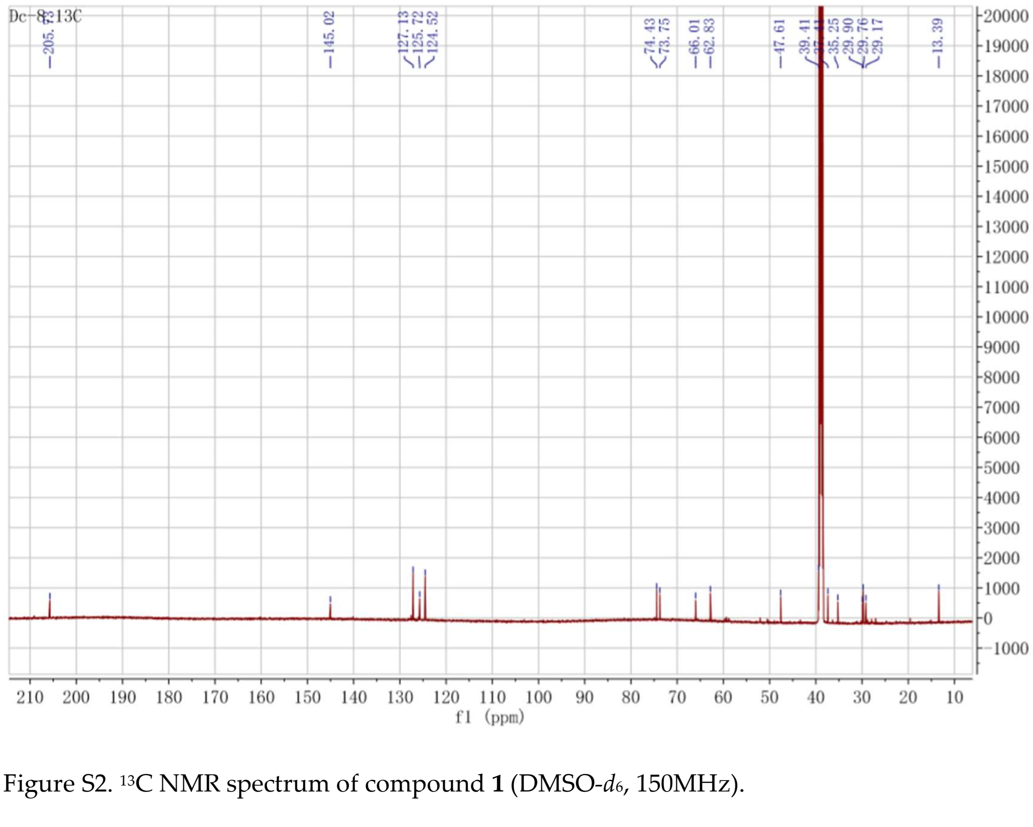

Figure S2. ^{13}C NMR spectrum of compound **1** (DMSO-d_6, 150MHz).

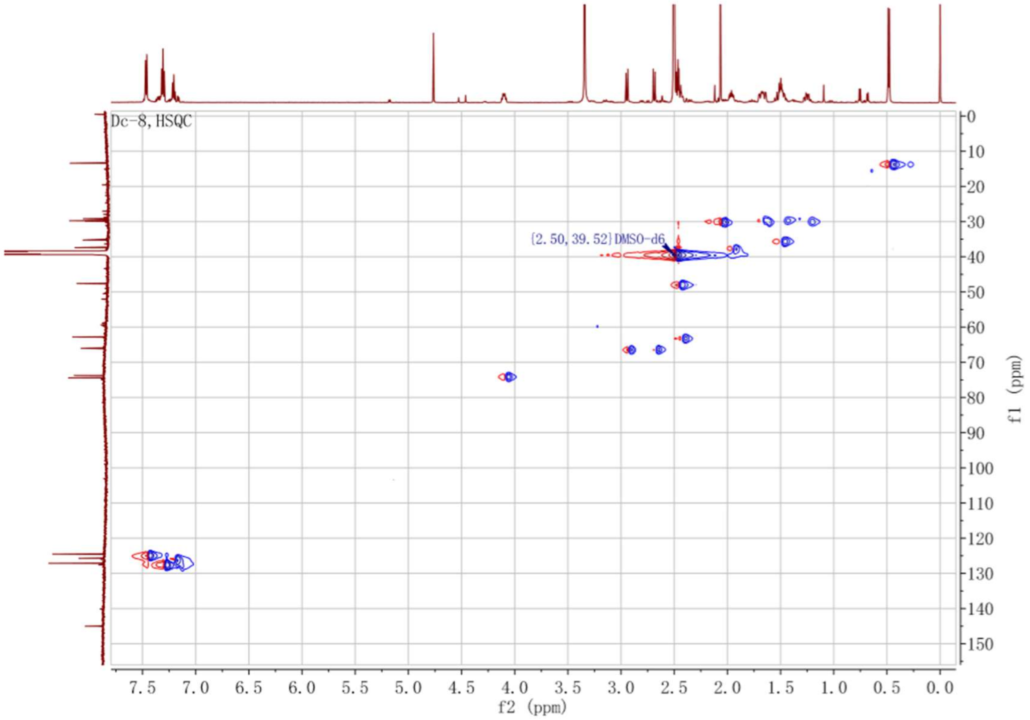

Figure S3. HSQC spectrum of compound **1** (DMSO-d_6, 600MHz).

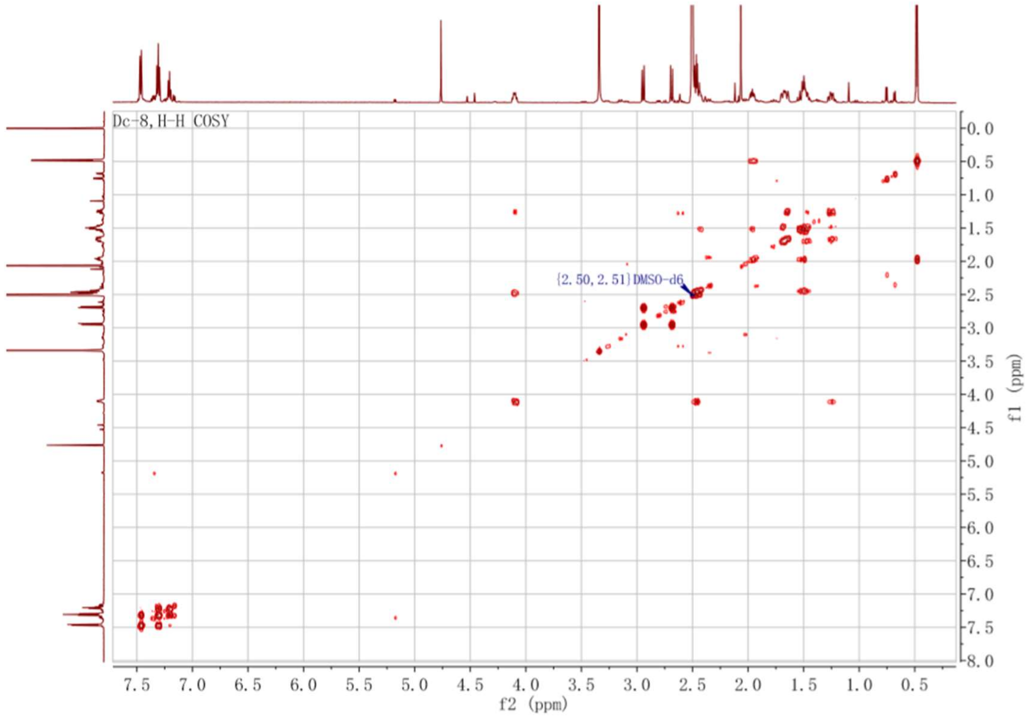

Figure S4. ^{1}H-^{1}H COSY spectrum of compound **1** (DMSO-d_6, 600MHz).

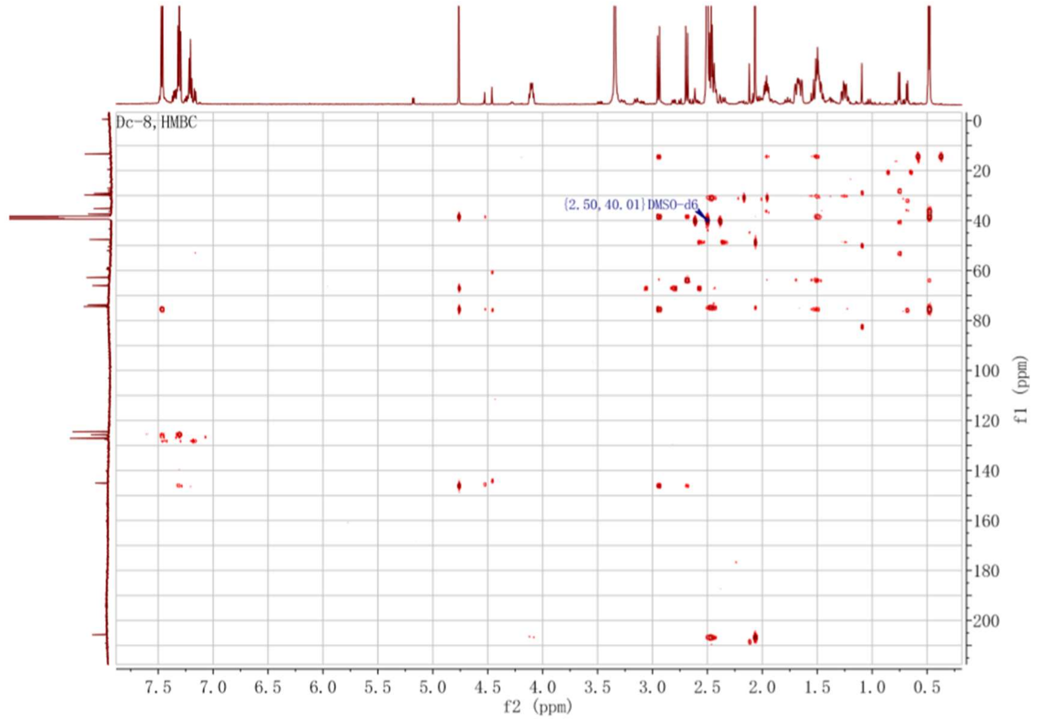

Figure S5. HMBC spectrum of compound **1** (DMSO-d_6, 600MHz).

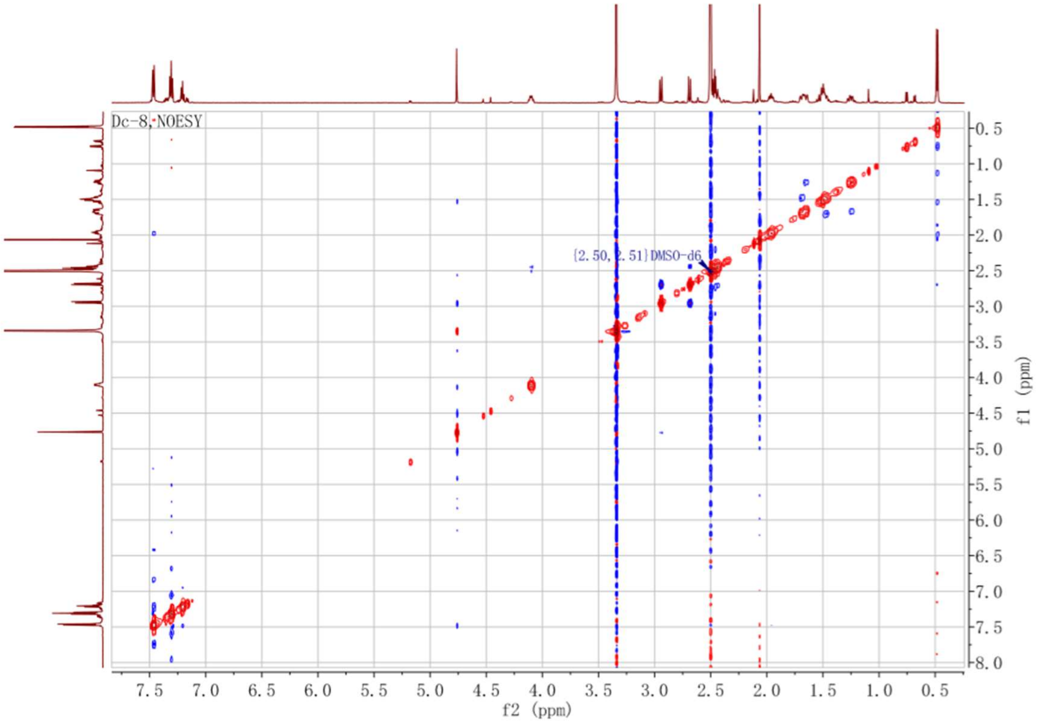

Figure S6. NOESY spectrum of compound **1** (DMSO-d_6, 600MHz).

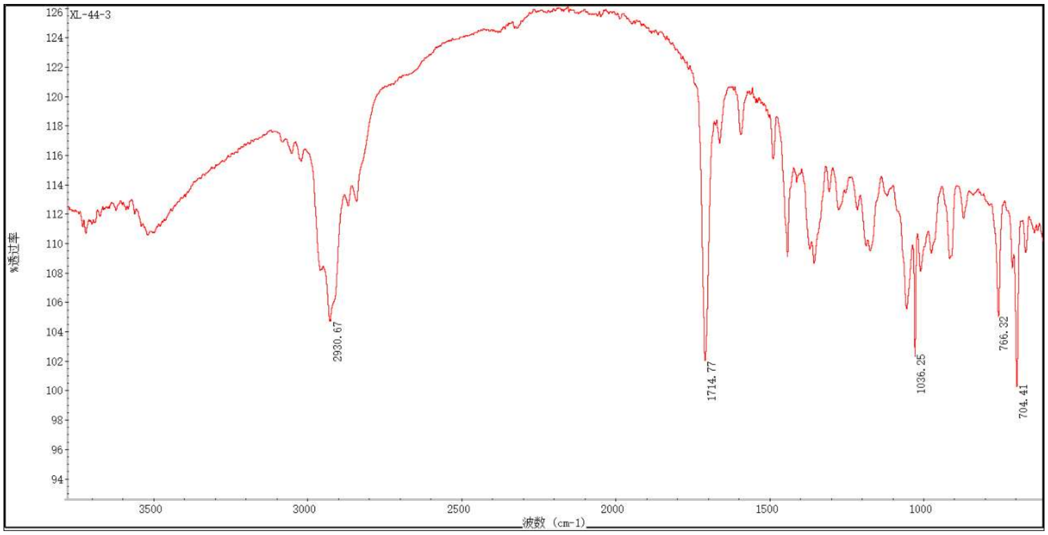

Figure S7. IR spectrum of compound **1.**

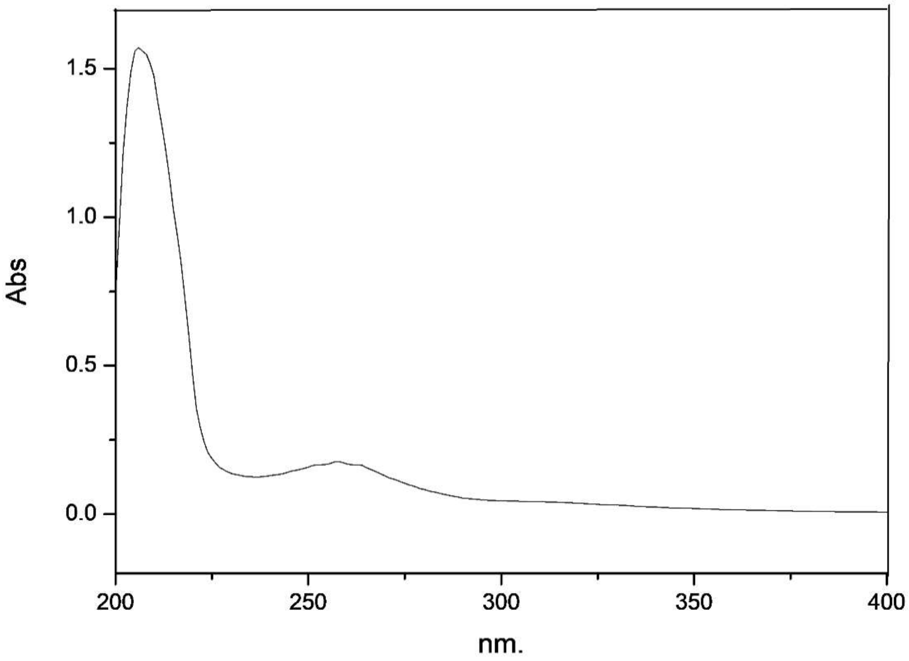

Figure S8. UV spectrum of compound **1** in CH_3OH.

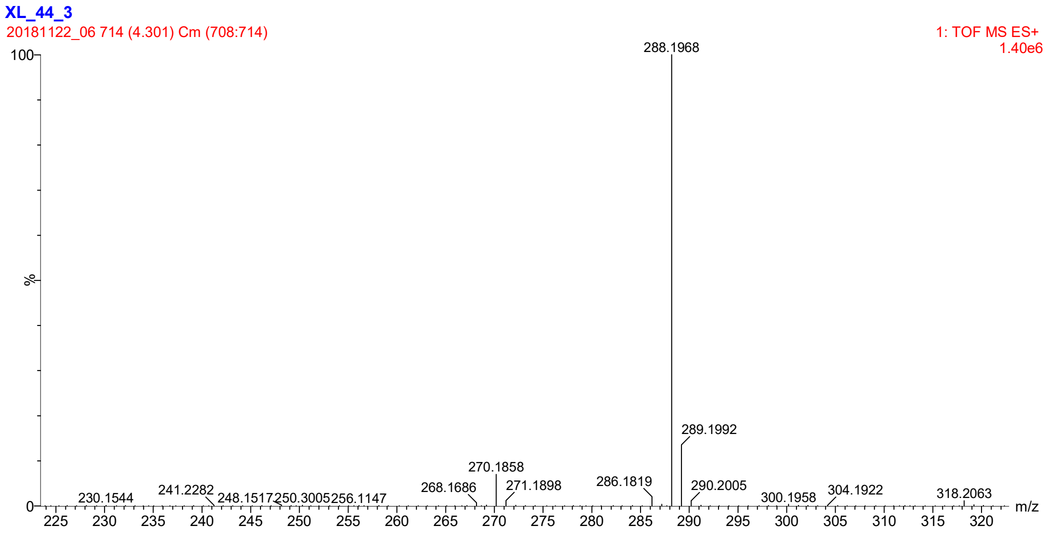

Figure S9. HRESIMS spectrum of compound **1**.

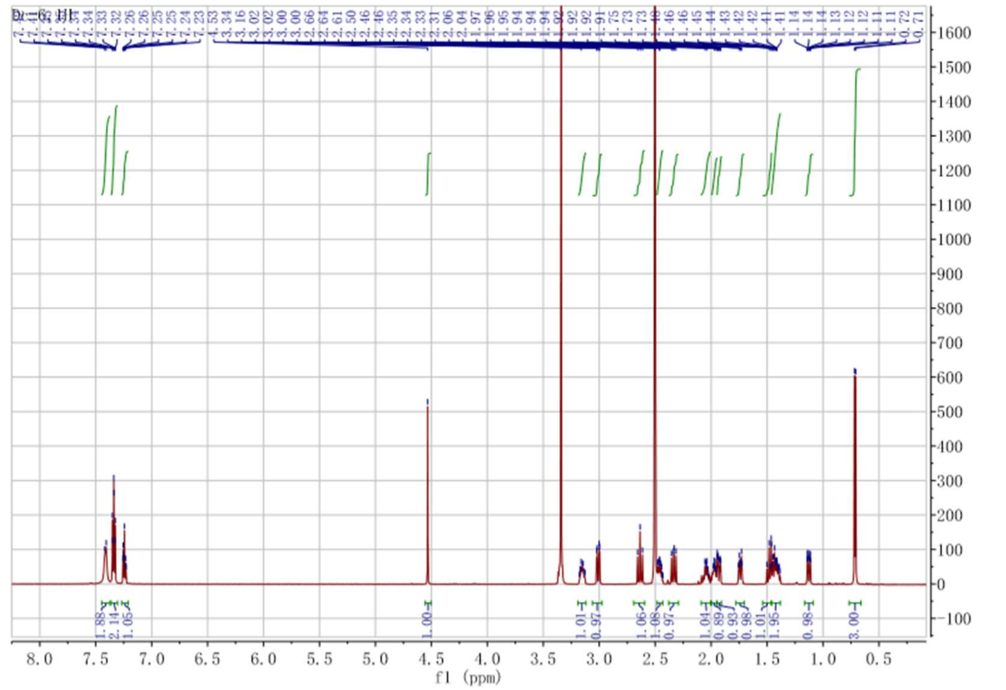

Figure S10. ^{1}HNMR spectrum of compound **2** (DMSO-d_6, 600MHz).

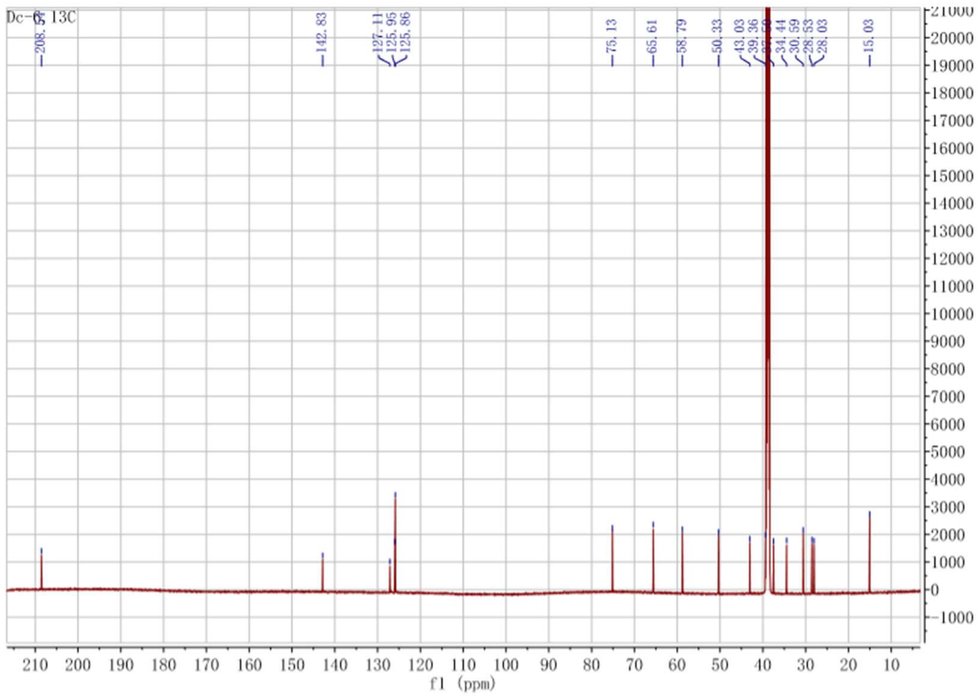

Figure S11. ^{13}C NMR spectrum of compound **2** (DMSO-d_6, 150MHz).

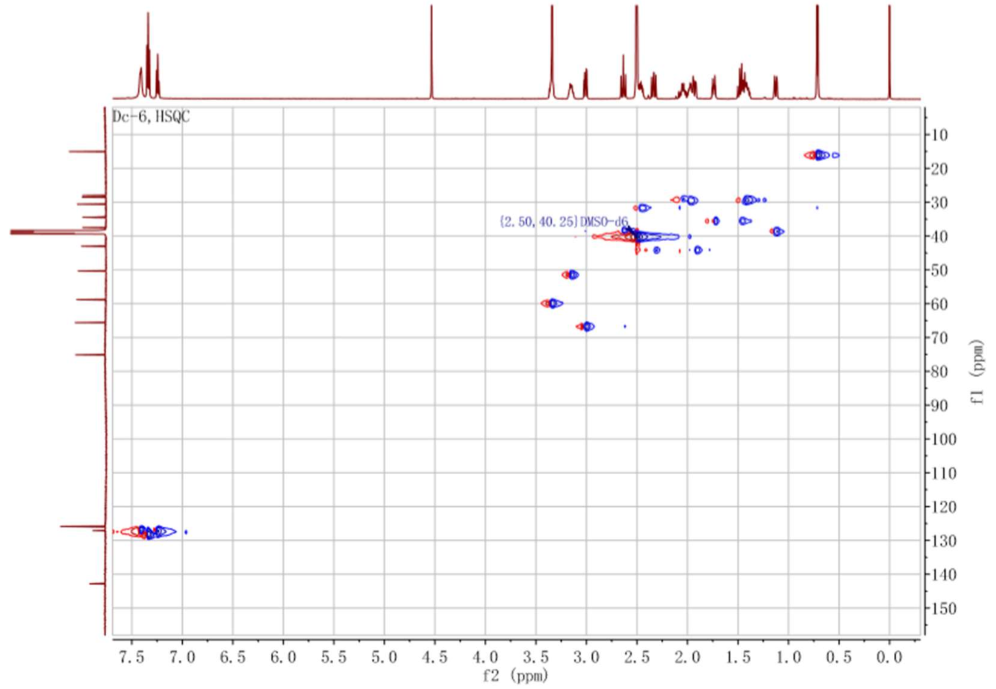

Figure S12. HSQC spectrum of compound **2** (DMSO-d_6, 600MHz).

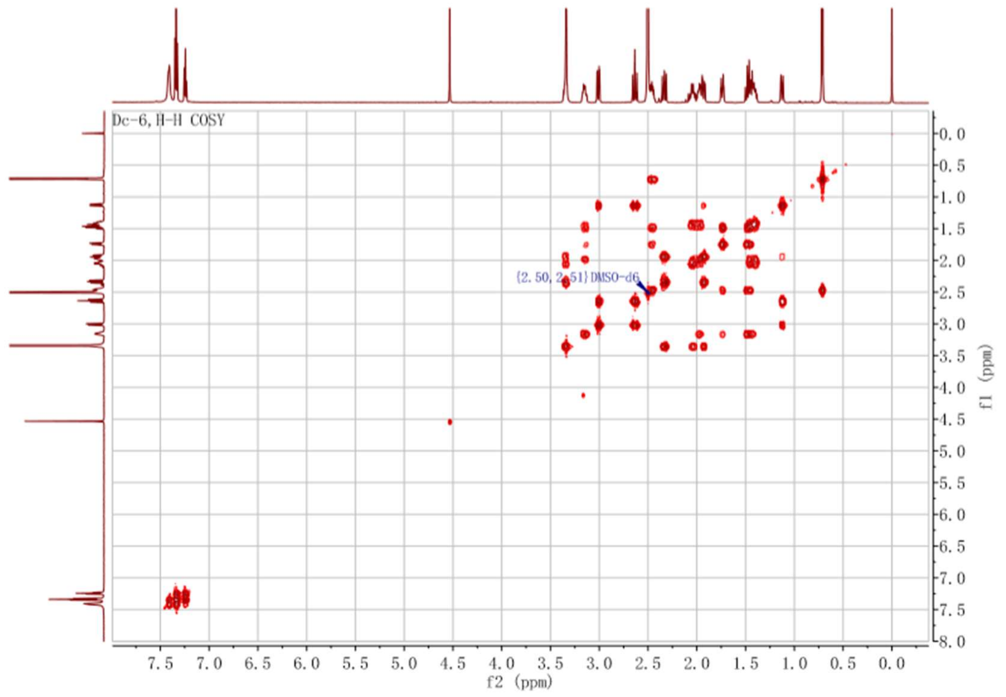

Figure S13. ^{1}H-^{1}H COSY spectrum of compound **2** (DMSO-d_6, 600MHz).

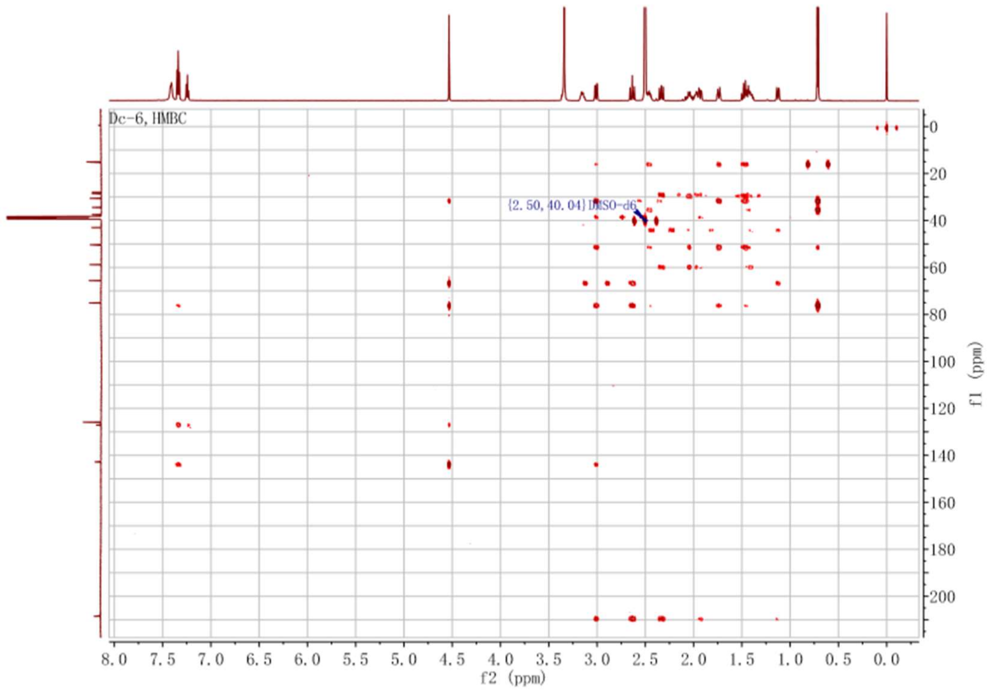

Figure S14. HMBC spectrum of compound **2** (DMSO-d_6, 600MHz).

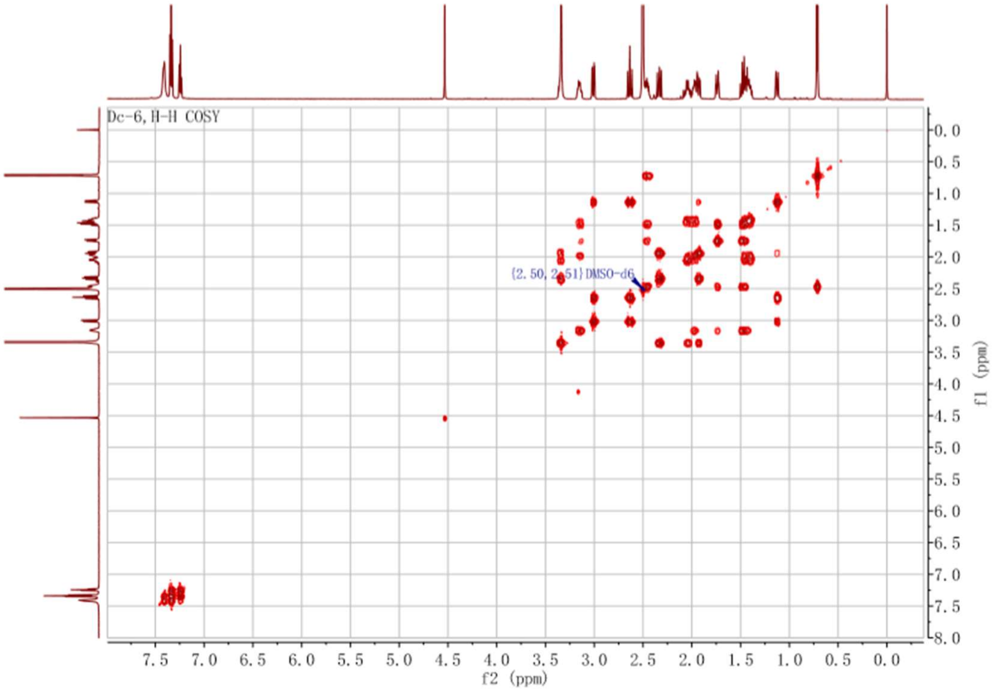

Figure S15. NOESY spectrum of compound **2** (DMSO-d_6, 600MHz).

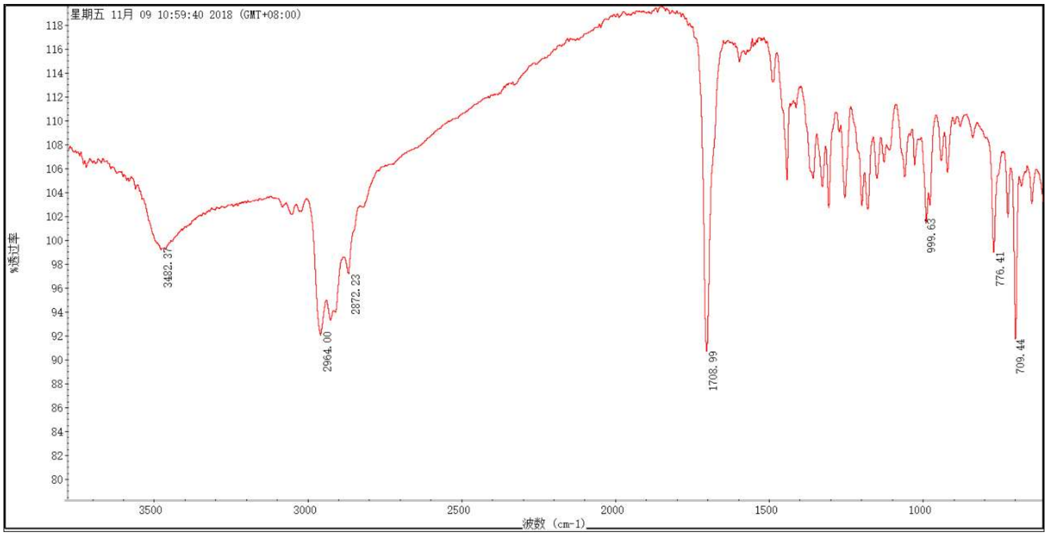

Figure S16. IR spectrum of compound **2.**

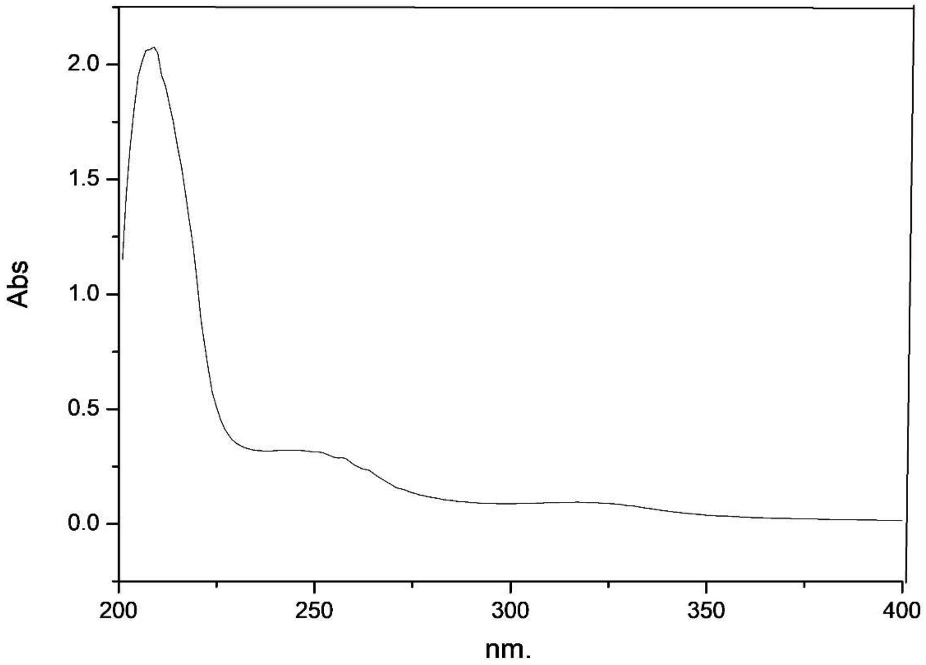

Figure S17. UV spectrum of compound **2** in CH3OH.

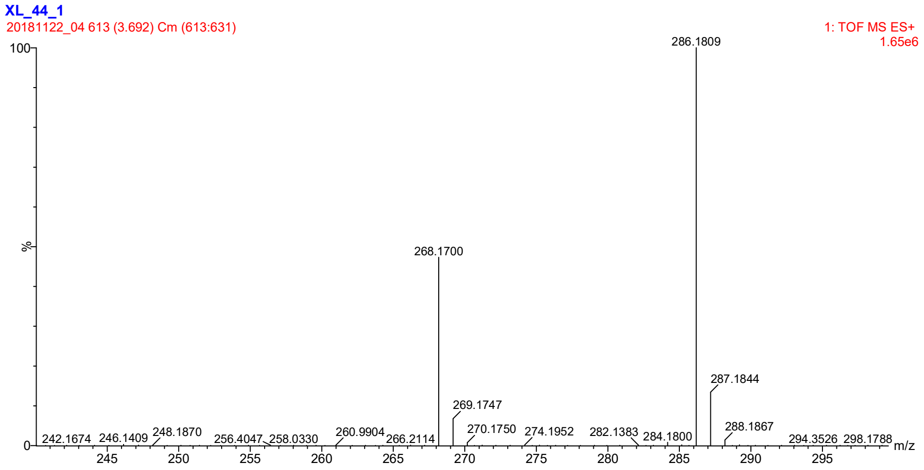

Figure S18. HRESIMS spectrum of compound **2**.
